# Does institutional quality matter for financial inclusion? International evidence

**DOI:** 10.1371/journal.pone.0297431

**Published:** 2024-02-02

**Authors:** Duc Hong Vo

**Affiliations:** Research Centre in Business, Economics & Resources, Ho Chi Minh City Open University Vietnam, Ho Chi Minh City, Vietnam; National University of Sciences and Technology, PAKISTAN

## Abstract

Financial inclusion is pivotal in supporting sustainable economic growth and social transformation. It is a key enabler for reducing poverty and uplifting prosperity. Improving financial inclusion has attracted significant attention from practitioners, academics, and governments. However, the asymmetric effect of institutional quality on financial inclusion contingent upon the income level has largely been neglected in the existing literature. As such, this study examines this asymmetric effect using the panel smooth transition regression for a sample of 110 countries globally from 2004 to 2020. Our empirical findings confirm the asymmetric effect of institutional quality on financial inclusion depending on the income level. Improved institutional quality is associated with extended financial inclusion in high-income and middle-income countries. However, low-income countries may not benefit from their institutional reform. Policy implications have emerged based on these empirical findings.

## Introduction

Financial inclusion has generally been considered a long and ongoing process connecting everyone to essential financial services. Financial inclusion has gained attention since the early 2000s because financial exclusion is directly linked with poverty. Financial institutions began transitioning from providing microcredit services to providing basic access to financial services. Financial inclusion has continuously asserted its pivotal role in sustainable economic growth and social transformation for countries globally. In recent years, financial inclusion has become an enabler for seven of 17 Sustainable Development Goals. The G20 countries reconfirm their commitment to global financial inclusion and implementing the G20 high-level principles for digital financial inclusion. Financial inclusion is a critical facilitator for reducing extreme poverty and increasing shared prosperity, which has set an ambitious target of achieving universal financial access in 2020 [1].

The significance of financial inclusion has drawn significant attention from scholars through its development journey. Financial inclusion, accompanied by the usage of mobile phones, promotes savings [2], reduces poverty, increases household consumption [3], and mitigates the costs of accessing financial services [4]. Financial inclusion reduces income inequality [5, 6] and CO_2_ emissions [7]. The existing literature has also addressed the obstacles to the development of financial inclusion, such as low institutional quality [8] and poor socio-characteristics [9]. As such, various studies have investigated the important role of institutional quality in financial inclusion in supporting financial development and economic growth [10–12] or as the facilitator of financial inclusion across countries [13–17].

The effects of institutional quality on financial inclusion have been extensively studied in the existing literature. However, the current literature has largely ignored how these effects change with different income levels. As such, this study separates itself from the others by incorporating the income effects when examining the impacts of institutional quality on financial inclusion across 110 countries from 2004 to 2020. The purpose of this study is to address the following three issues: (i) the extent to which the government’s decisions impact financial inclusion, (ii) whether countries with better institutional quality exhibit a greater level of financial inclusion, and (iii) whether an asymmetric effect of institutional quality on financial inclusion exists. As such, the contributions of this study to the existing literature are threefold. *First*, we contribute to the body of knowledge regarding the impacts of institutional quality on financial inclusion using macroeconomic fundamentals, social characteristics, and institutions in a cross-country context. *Second*, we examine the moderating effect of income level on the institutional quality–financial inclusion nexus. *Third*, we advocate using the panel smooth transition regression (PSTR) model, which is considered a superior alternative method compared to conventional estimation methods such as the fixed and random effects estimation, the quantile regression and the threshold estimator.

Following this introduction, the remainder of this paper is structured as follows. Literature review section synthesizes the related studies on financial inclusion to identify the research gap. Data and research methodology section presents the empirical strategy and data. Empirical results are presented and discussed in Findings and discussions section, followed by the Conclusions and Policy Implications section.

## Literature review

The early study of Leyshon and Thrift [18] laid the foundation for developing the current body of knowledge on financial inclusion. Particularly, their study addresses the aspects that hinder certain classes of society from accessing the formal financial system, the so-called financial exclusion. Since then, various studies have provided different definitions of financial exclusion under different contexts, such as Carbó [19], Conroy [20] and Sinclair [21]. However, these definitions present a broadly general approach to financial inclusion for societal classes. Sarma [22] puts forward a comprehensive definition of financial inclusion that considers all participants of an economy and incorporates different dimensions of financial inclusion. In particular, Sarma [22] initiates and estimates the index of financial inclusion (IFI) as a comprehensive measure of the degree of financial inclusion. The index has significantly gained its practicability. The IFI has been widely adopted until now.

Various scholars have examined the critical role of financial inclusion in various aspects of social prosperity. Ouma et al. [2] investigate the effects of the widespread usage of mobile phones to offer financial services on savings mobilization in Sub-Saharan African nations. These findings suggest that using mobile phones to deliver financial services increases savings at home. Abor et al. [3] use a large sample of Ghanaian families to study the welfare consequences of mobile telephony’s multifunctional nature. The findings suggest that having mobile phones and access to financial services reduces the likelihood of becoming impoverished and increases per capita household consumption of food and non-food products. Gebrehiwot and Makina [4] suggest that mobile technology plays a positive and promising role in boosting financial inclusion. Mobile phone penetration may be accomplished at a lower cost, accelerating attempts to attain financial inclusion compared with bank branches’ penetration,

Erlando et al. [6] examine the role of financial inclusion on economic growth, poverty reduction, and income inequality in Eastern Indonesia. The results show that financial inclusion, economic growth, poverty, and income distribution have all been linked. Financial inclusion increases because of socioeconomic progress, while poverty decreases. Financial inclusion, on the other hand, reduces income inequality, resulting in a more balanced income distribution in Eastern Indonesia. This finding aligns with Demir et al. [5], who investigated the relationship between fintech, financial inclusion, and income inequality for 140 countries in 2011, 2014 and 2017. Findings indicate that fintech indirectly reduces income inequality via its effects on financial inclusion. Financial inclusion reduces income inequality at all different quantiles of income. Financial inclusion reduces income inequality, particularly in high-income nations. On a different angle, Renzhi and Baek [7] examine whether financial inclusion can be considered an effective mitigation measure against greenhouse gas emissions for 103 countries from 2004 to 2014. The empirical results reveal an inverted U-shaped relationship between financial inclusion and CO_2_ emissions. These findings confirm the validity of the environmental Kuznets curve in these countries. Interestingly, the findings imply that different phases of financial inclusion impact differently on CO_2_ emissions levels.

However, problems hindering the development of financial inclusion are also observed. These main roadblocks may be classified into four categories: (i) social, macroeconomic, and infrastructure features, (ii) institutional quality, (iii) banking-related obstacles, and (iv) regulatory distortion. Love and Peria [23] consider that two major factors drive financial inclusion. First, the structural factors primarily determine the cost of providing financial services to the community. Second, policy-related factors are important in providing an enabling environment for financial inclusion. Several studies have addressed certain obstacles to the determinants of financial inclusion. Andrianova et al. [8] present a credible explanation for the underdevelopment of the African banking sector due to a poor credit market. The underdeveloped credit markets in African countries are caused by a moral hazard problem (due to strategic loan defaults) or potentially an adverse selection problem (emanating from a lack of viable investment projects). The findings imply that when institutional quality is low, loan defaults are a key factor constraining bank lending. Ghosh and Vinod [9] investigate the culprit that makes it difficult for women to access financial inclusion by looking at whether gender plays a role in financial inclusion and, if so, the factors that may impact this link. The results indicate that there is a large gender gap in both access to and usage of financial services and products. Also, education and earnings are more important in explaining access to financial services and products for female-headed households. In contrast, political and social factors are more important in explaining differences in financial usage.

Recognizing the important role of government in advancing financial inclusion, many scholars have focused on institutional quality as an effective tool in improving financial inclusion across countries globally, especially in emerging economies. Institutional quality is a broad term encompassing legal principles, individual rights, and high-quality government regulation and services. Different determinants that play the most critical role in enhancing financial inclusion have been selected from several studies. Eldomiaty et al. [14] examine the effect of world governance indicators (WGIs) on the amelioration of financial inclusion in different countries. Their findings reveal that government effectiveness, political stability, voice, and accountability are the primary financial services and products that significantly impact financial inclusion. Using a broader approach, Ozili [17] examines the circumstances of financial inclusion in various nations and regions, focusing on the African, Asian, European, and American regions. The empirical results indicate that financial inclusion impacts and is impacted by the extent of financial innovation, poverty reduction, financial sector stability, the status of the economy, financial literacy, and regulatory frameworks.

Previous studies have also examined the effect of institutional quality on financial inclusion for emerging and advanced economies. Zulkhibri and Ghazal [12] investigate the relationship between financial inclusion, institutions, and governance in Muslim countries and developing economies. They find that governance positively affects financial inclusion by increasing the number of bank accounts and savings in formal financial institutions. There are also considerable disparities in understanding financial inclusion among countries and regions. Ali et al. [11] investigate the moderating relationship between financial inclusion and institutional quality for 45 countries in the Organization of Islamic Cooperation (OIC). The empirical findings reveal that financial inclusion, institutional quality, and financial development are positively linked. Surprisingly, institutional quality reduces financial inclusion and provides a major beneficial influence on financial development. Ahmed et al. [10] confirm the significance of institutional quality and financial development on green economic growth in South Asian nations from 2000 to 2018. The findings show that institutional quality and financial development are important for long-term green economic success.

Ongo Nkoa and Song [16] studied the effects of institutional quality on financial inclusion in 51 African countries. The findings suggest that institutional quality boosts financial inclusion and financial service penetration, accessibility, and use in Africa. Aracil et al. [13] examined the role of institutional quality in the link between financial inclusion and poverty reduction in a sample of 75 developing and developed countries from 2004 to 2017. The findings imply that institutional quality affects the relationship between financial inclusion and poverty. With high institutional quality, financial inclusion significantly impacts poverty reduction. The impact is even more significant in the poorer nations. Muriu [15] examines the effects of institutional quality on financial inclusion at the bank level in developed and emerging nations. The results indicate that a better enabling environment for accessing financial services, specifically the rule of law, relates to increased financial inclusion.

Our literature review indicates that while the effects of institutional quality on financial inclusion have been extensively investigated, the moderating role of income on these effects has largely been neglected in the existing literature. This review warrants our study to be conducted to provide additional evidence on the asymmetric effects of institutional quality on financial inclusion when income is considered across countries globally.

## Data and research methodology

### A research framework

Institutional quality contains three main divisions: (i) regulation, (ii) law enforcement, and (iii) macroeconomic conditions. We use these three aspects of institutional quality as our analytical framework, focusing on the following research questions: (i) To what extent does governance affect financial inclusion? (ii) Does a country with better institutional quality have a high degree of financial inclusion? Furthermore, (iii) Does the institutional quality have any asymmetric effect on financial inclusion, depending on the income level? Each of these research questions is responded to below.

*First*, countries with an effective legal system likely have a broad credit supply and demand base. As such, the country will probably exhibit a large proportion of their economic agents participating in the financial market [12, 15, 24]. A country with an efficient legal system, such as tax policy and enterprise law, will motivate foreign and domestic investors/corporates to conduct business. *Second*, trade liberalization and deregulation will exert an upward effect on financial inclusion because they reduce the excessive procedural processes involved in financial product usage. Furthermore, deregulation is anticipated to save public resources. Likewise, reducing regulatory stringency also fosters the development and adaptation of higher financial technologies, such as digital banking, E-wallets, and various means of online payment. *Third*, political stability acts as a prerequisite for financial inclusion. Andrianova et al. [8] and Muriu [15] consider that contractual enforcement mitigates the unwanted externality of financial inclusion in terms of provision for non-performing loans, resulting from information asymmetry between lenders and borrowers.

### Measuring financial inclusion

We follow the approach of estimating the index of financial inclusion (IFI) from Camara and Tuesta [25] and Ahamed and Mallick [26], using the principal components analysis (PCA). Table 1 presents the estimates of the index of financial inclusion (IFI) for 110 countries globally. The IFI index is normalized to the range of 0 to 1. The higher the IFI estimate, the more financially inclusive the country.

**Table 1:** The estimates of the index of financial inclusion (IFI) for 110 countries.

Country	IFI	Country	IFI	Country	IFI
Afghanistan	0.11	Guinea	0.02	Mauritius	0.38
	(0.01)		(0.02)		(0.04)
Angola	0.14	Greece	0.35	Malaysia	0.38
	(0.03)		(0.03)		(0.03)
Albania	0.22	Honduras	0.22	Namibia	0.28
	(0.04)		(0.02)		(0.08)
United Arab Emirates	0.40	Croatia	0.34	Nicaragua	0.14
	(0.08)		(0.05)		(0.02)
Argentina	0.15	Hungary	0.23	Netherlands	0.34
	(0.03)		(0.02)		(0.03)
Armenia	0.17	Indonesia	0.18	Norway	0.26
	(0.08)		(0.04)		(0.02)
Australia	0.49	India	0.20	Nepal	0.22
	(0.04)		(0.03)		(0.05)
Austria	0.15	Ireland	0.39	Pakistan	0.19
	(0.03)		(0.08)		(0.01)
Azerbaijan	0.13	Iran, Islamic R	0.24	Panama	0.44
	(0.03)		(0.06)		(0.04)
Belgium	0.35	Iraq	0.14	Peru	0.17
	(0.03)		(0.01)		(0.07)
Bangladesh	0.15	Iceland	0.45	Philippines	0.14
	(0.02)		(0.08)		(0.03)
Bulgaria	0.28	Italy	0.37	Poland	0.26
	(0.06)		(0.03)		(0.04)
Bosnia and Herzegovina	0.27	Jamaica	0.17	Portugal	0.56
	(0.02)		(0.02)		(0.05)
Bolivia	0.19	Jordan	0.38	Palestinian Territory	0.22
	(0.06)		(0.01)		(0.03)
Brazil	0.31	Japan	0.44	Romania	0.27
	(0.01)		(0.03)		(0.02)
Brunei	0.23	Kazakhstan	0.26	Russia	0.31
	(0.03)		(0.03)		(0.09)
Botswana	0.18	Kenya	0.11	Rwanda	0.07
	(0.01)		(0.02)		(0.02)
Switzerland	0.55	Cambodia	0.16	Saudi Arabia	0.23
	(0.06)		(0.09)		(0.04)
Chile	0.28	Korea, Rep	0.58	Singapore	0.48
	(0.03)		(0.05)		(0.05)
China	0.44	Kuwait	0.29	El Salvador	0.23
	(0.05)		(0.05)		(0.01)
Cameroon	0.05	Lao PDR	0.13	San Marino	0.88
	(0.01)		(0.04)		(0.1)
Colombia	0.19	Lebanon	0.53	Serbia	0.22
	(0.03)		(0.03)		(0.03)
Comoros	0.06	Lesotho	0.10	South Sudan	0.06
	(0.01)		(0.02)		(0.01)
Cape Verde	0.31	Lithuania	0.24	Slovakia	0.25
	(0.05)		(0.02)		(0.03)
Costa Rica	0.25	Luxembourg	0.33	Slovenia	0.36
	(0.03)		(0.03)		(0.04)
Cyprus	0.44	Latvia	0.31	Sweden	0.25
	(0.04)		(0.05)		(0.02)
Czech Republic	0.26	Moldova	0.24	Seychelles	0.27
	(0.03)		(0.02)		(0.03)
Germany	0.38	Madagascar	0.05	Chad	0.02
	(0.01)		(0.01)		(0.01)
Denmark	0.30	Maldives	0.21	Thailand	0.33
	(0.05)		(0.01)		(0.04)
Dominica Republic	0.13	Mexico	0.15	Trinidad	0.23
	(0.02)		(0.02)		(0.03)
Ecuador	0.17	Macedonia	0.24	Turkey	0.25
	(0.02)		(0.05)		(0.05)
Egypt	0.20	Malta	0.42	Uganda	0.10
	(0.01)		(0.02)		(0)
Spain	0.60	Myanmar	0.07	Ukraine	0.24
	(0.1)		(0.03)		(0.04)
Estonia	0.33	Montenegro	0.30	Vietnam	0.33
	(0.04)		(0.05)		(0.05)
Finland	0.23	Mongolia	0.30	Zambia	0.09
	(0.02)		(0.07)		(0.01)
Georgia	0.21	Mozambique	0.09	Zimbabwe	0.21
	(0.09)		(0.03)		(0.07)
Ghana	0.20	Mauritania	0.56		
	(0.01)		(0.12)		

Our estimates of the financial inclusion index indicate that high-income countries appear to have a higher degree of financial inclusion. The top five countries achieving the highest degree of financial inclusion include San Marino (0.88), Spain (0.6), South Korea (0.58), Mauritania (0.56), and Portugal (0.55). In contrast, Guinea, Chad, Cameroon, Madagascar, and Comoros have achieved the lowest financial inclusion among 110 countries in the sample.

### Model specification

Our empirical model examining the effects of institutional quality on financial inclusion for 110 countries globally is expressed as follows:

IFI=f(institutions,macroeconomics,sociocharacteristics)

where IFI stands for the level of financial inclusion.

- *Institutions* representing the institutional quality are proxied by three indicators, including (i) *government effectiveness*, (ii) *rule of law*, and (iii) *regulatory quality*. These indicators are composite indices whose values vary between -2.5 and 2.5. Data on these indices are collected from the Worldwide Governance Indicators (WGI). This WGI dataset provides the annual assessment for 200 countries.
- *Macroeconomics* are proxies for the heterogeneity in macroeconomic conditions, such as inflation rate, income level, and GDP per capita. Data for these variables are collected from the World Development Indicators (World Bank).
- *Socio characteristics* include urbanization, which is the ratio between the urban population and the total population. The data is also available from the World Bank database.

### The panel smooth transition regression

Our empirical model is expressed below:

$$Y_{it}=\mu +\beta_{0}Z_{it}+\alpha_{0}X_{it}+\varepsilon_{it}$$ (1)

Where *Y*_*it*_ represent the financial inclusion of country *i* in time *t*. *X*_*it*_ denotes the matrix of the explicative vectors, and *Z*_*it*_ is our variable of interest—the proxies for institutional quality. Our sample includes 110 countries globally. As such, expecting one homogenous coefficient *β*_0_ for every country is relatively impractical. In other words, unobserved factors change the effects of institutional quality on financial inclusion across countries differently.

The panel smooth transition regression (PSTR) was first introduced in the seminal work by González et al. [27]. This estimator is generally considered superior to the panel threshold regression since the estimation allows the transitions around the thresholds. Colletaz and Hurlin [28] argue that the PSTR estimator allows the extent and direction of explanatory vectors to be heterogeneous based on the whereabouts of *y*_*it*_ on the function *f*(*y*_*it*_, *γ*, *c*). Another advantage of the PSTR estimator is that the method can also remove the time-persistent characteristics by incorporating fixed and random effect estimators into the within-regime regression. In sum, Eq (1) with two regimes becomes:

$$Y_{it}=\mu_{i}+\beta_{0}Z_{it}+\alpha_{0}X_{it}+(\beta_{1}Z_{it}+\alpha_{1}X_{it})\times f(GDP_{it},\gamma ,c)+\varepsilon_{it}$$ (2)

Where: *X*_*it*_ denotes a matrix of control variables. *Z*_*it*_ denotes vectors for institutional quality. *γ* indicates how fast the vector of coefficients changes concerning the changes in *GDP*_*it*_. *c* denotes the threshold at which the transition begins. *β*_0_, *β*_1_, *α*_0_, *and α*_1_ are coefficients of the explicative vectors belonging to the first regime and second regime, respectively. In our model, we consider that the GDP per capita is a time-variant factor that induces the difference and inconsistency of the impact of institutional quality on financial inclusion. *ε*_*it*_ is the residual with a mean value of 0 and the variance of *σ*^2^. *f*(*GDP*_*it*_, *γ*, *c*) represents the transitive function, which contains the information about where and how the countries from the lower-income group move up to the higher-income group. The *f*(*GDP*_*it*_, *γ*, *c*) are mathematically expressed and graphically as below:

$$f(GDP_{it},\gamma ,c)={\left[1+exp(-\gamma \prod_{z=1}^{m}\left(GDP_{it}-c_{z}\right))\right]}^{-1},\;\gamma >0;\;c_{1}\leq c_{2}\leq \cdots \leq c_{m};$$

*Where*: *m* is the number of regimes whose determinations are demonstrated below.

**Fig 1:**
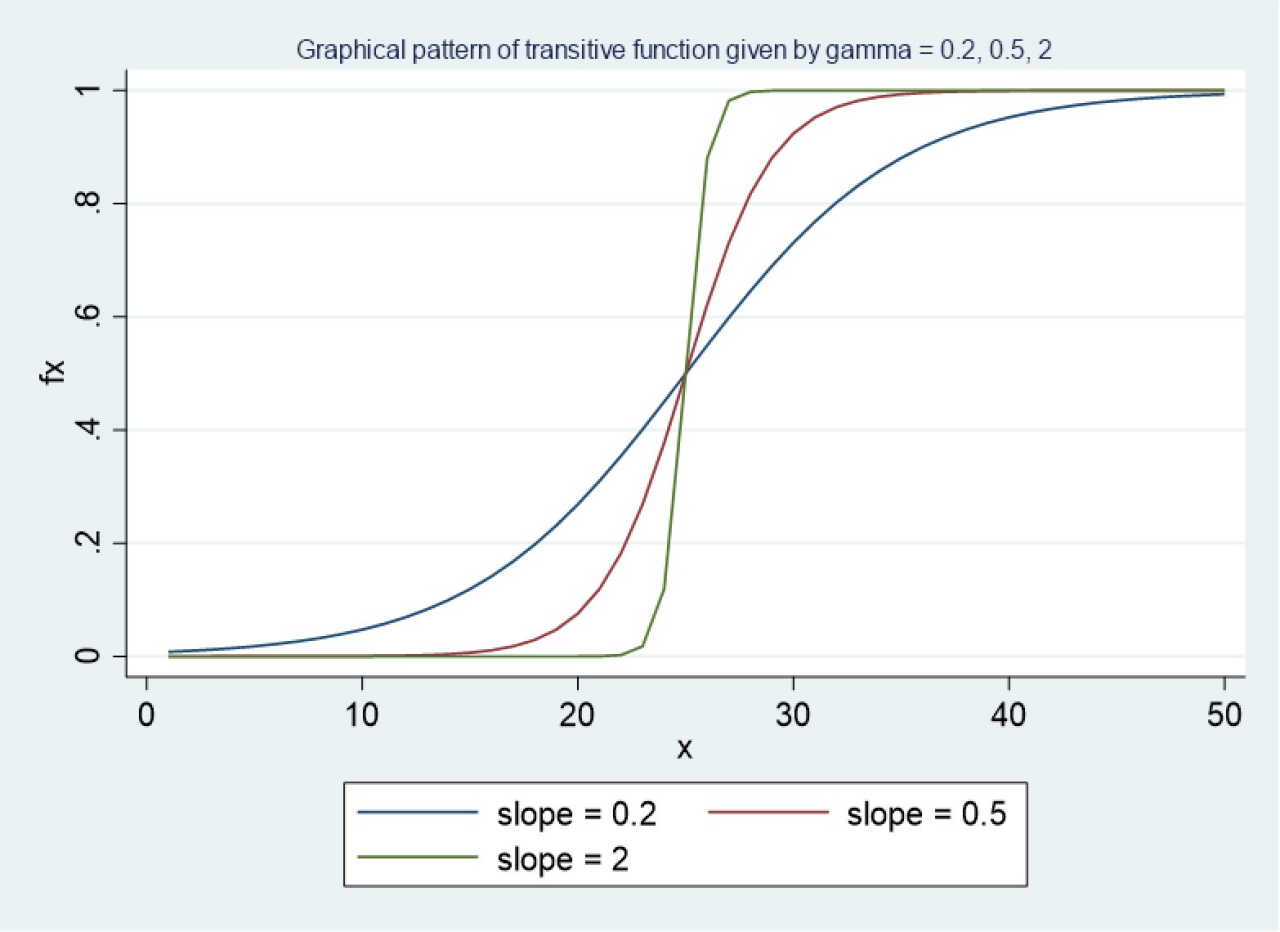
The graphical presentation of the transition function with *γ* = 0.2, 0.5, *and* 2.

Fig 1 indicates that the impacts from the right-hand-sided variables on *y*_*it*_ in one country are equal to the sum of *β*_0_+*β*_1_×*f*(*GDP*_*it*_, *γ*, *c*) since 0≤*f*(*GDP*_*it*_, *γ*, *c*)≤1. Let us denote the marginal effects as *e*, and then the marginal effect or elasticity of institutional quality is expressed as $e_{it}^{INS}=\frac{\partial y_{it}}{\partial INS}=\beta_{0}+\beta_{1}\times f(GDP_{it},\gamma ,c)$. The range in which the random variable of elasticity *e*^*INS*^ varies is defined as: *β*_0_≤*e*^*INS*^≤*β*_0_+*β*_1_ if *β*_1_≥0 or *β*_0_+*β*_1_≤*e*^*INS*^≤*β*_0_ if *β*_1_≤0. To eliminate the unobserved time-invariant factors and capture the change of time-variant factors, the first step is to subtract the country-wide means of the included variables to account for the fixed effect. The process is straightforward with the proxy of financial inclusion: ${\tilde{y}}_{it}=y_{it}-{\bar{y}}_{i}$. However, the process becomes complicated with the matrix of explicative variables *X* because their values depend on the function *f*(*GDP*_*it*_,*γ*,*c*). The transformation corresponding to the first regime, *β*_0_, is identical to the dependent variable ${\tilde{X}}_{it}=X_{it}-{\bar{X}}_{i}$. Values of *X* in successive regimes are demeaned by multiplying the value of the transitive function by the transformation: $X_{it}=X_{it}f(GDP_{it},\gamma ,c)-\frac{1}{T}\sum_{t=1}^{T}f(GDP_{it},\gamma ,c)$. After removing the unobserved time-invariant fixed effects, the coefficients $\beta_{0},\beta_{1},\alpha_{0}$ and *α*_1_ can be derived using the ordinary least square, which is expressed as $\beta^{*}={\left({\tilde{X}}^{\prime }\tilde{X}\right)}^{-1}{\tilde{X}}^{\prime }\tilde{y}$, where β* = *β*_0_, *β*_1_, *α*_0_, *α*_1_ is the matrix of the estimated coefficients. The example above represents the model’s generalization with only one turning point, c that divides the sample into two regimes. It is worth noting that the entire process can be replicated for the different number of regimes (i.e., *m* = 3,4,…,*n*.)

We assume that the impact of institutional quality on financial inclusion is asymmetric. We follow the testing procedure outlined in González et al. [27]. Beginning with the assumption of linearity, we examine the validity of the null hypothesis of *H*_0_:*γ* = 0 versus the alternative of *H*_1_:*γ*≠0. The Lagrange multiplier test statistic and its F-version are expressed as follows:

$$LM=\frac{TN(SSR_{0}-SSR_{1})}{SSR_{0}}$$

$$LMF=\left[\frac{SSR_{0}-SSR_{1}}{Km}\right]/\left[\frac{SSR_{0}}{TN-N-mK}\right]$$

$$LRT(m)∼\chi^{2}(mK)=-2[\log\left(SSR_{1}\right)-log(SSR_{0})]$$

where *K* is the number of regressors. *m* is the number of regimes. *SSR*_0_ and *SSR*_1_ are the sum squared residuals of the models corresponding to the linear and regime-switching models. *T* and *N* are the time and cross-sectional dimensions of the sample. If the test result fails to support the null hypothesis, it implies that the asymmetric effect exists. Finally, we reapply the test until the null hypothesis *H*_0_ cannot be rejected.

## Findings and discussions

### Preliminary tests

The pairwise correlations between variables used in our analysis are estimated to ensure that no potential multicollinearity exists in our models. Multicollinearity might not affect the unbiasedness of the estimators. However, it may affect our conclusions on accepting or rejecting the estimated coefficients. The correlation matrix and variance inflation factor (VIF) are presented in Table 2 below.

**Table 2:** The pairwise correlation and VIF of the variables.

Variables	VIF	(1)	(2)	(3)	(4)	(5)	(6)	(7)
(1) Financial inclusion	.	1.000						
(2) Inflation	1.17	-0.311*	1.000					
(3) GDP per capita	3.27	0.667*	-0.351*	1.000				
(4) Urban population	1.02	0.015	0.108*	-0.029	1.000			
(5) Rule of law	2.79	0.599*	-0.356*	0.793*	-0.115*	1.000		
(6) Government Effectiveness	3.30	0.622*	-0.370*	0.833*	-0.049*	0.958*	1.000	
(7) Regulatory quality	2.92	0.575*	-0.390*	0.812*	-0.078*	0.934*	0.941*	1.000

Notes

* denotes the significance at 10 per cent.

Table 2 confirms that the VIF is below four, implying no evidence of multicollinearity. In addition, the PSTR estimation used in this study is a static model. Non-stationarity reduces the reliability of the estimated coefficients. As such, we extend the preliminary tests by examining the non-stationarity for the demeaned series using the first- and second-generation unit root tests. Dong et al. [29] argue that the first-generation unit root test is disadvantaged and impractical by the assumption of homogeneity and uncorrelation among individuals. As such, the second-generation unit root tests are more appropriate. However, we present empirical results from the first- and second-generation unit root tests in Table 3 below for completeness.

**Table 3:** Empirical results from the first- and second-generation unit root tests.

	IFI	GDPP	INF	POP	ROL	GOE	REQ
IPS	1.573	0.411	-13.673***	-32.665***	-1.368*	-4.598***	-2.364***
	(0.942)	(0.660)	(0.000)	(0.000)	(0.086)	(0.000)	(0.009)
CADF	2.094	3.805	-11.064***	4.175	-3.675***	-7.909***	-6.643***
	(0.982)	(1.000)	(0.000)	(1.000)	(0. 000)	(0.000)	(0.001)

Notes

*, **, and *** denote the statistical significance at 10, 5, and 1 per cent. The null hypothesis of IPS and Pesaran’s cross-sectional unit root test under cross-sectional dependency assumes that the entire panel contains a unit root. The alternative hypothesis is that at least one series is stationary. The p-values are reported in parentheses. **ROL**—The rule of law; **GOE**—Government effectiveness; **REQ**—Regulatory quality.

Empirical evidence from Table 3 confirms the non-stationarity of financial inclusion and income. The PSTR estimator is developed to improve the consistency and reliability of the estimated coefficients in the context of non-stationary problems [28]. Adopting the PSTR estimator to examine the asymmetric effect of institutional quality on financial inclusion is appropriate.

### Empirical results on the asymmetric effects of institutional quality on financial inclusion

We first substantiate the assumption of asymmetric effects and the possible number of regimes by reporting the results from slope homogeneity tests developed by Pesaran and Yamagata [30]. The test statistics are reported in Table 4 below:

**Table 4:** The slope homogeneity test and the linearity test.

	The slope homogeneity test (Pesaran and Yamagata test)		
	**The rule of law**	**Government effectiveness**	**Regulatory quality**
Δ	21.551***	23.418***	19.553***
	(0.000)	(0.000)	(0.000)
Δ_*adj*_	28.018***	30.445***	25.395***
	(0.000)	(0.000)	(0.000)
	**Linearity test (H**_**0**_ **: r = 0, H**_**a**_ **: r = 1)**		
LM	35.519	26.092	38.288
	(0.000)	(0.000)	(0.000)
LMF	8.454	6.174	9.130
	(0.000)	(0.000)	(0.000)
LRT	35.951	26.324	38.790
	(0.000)	(0.000)	(0.000)
	**Linearity test (H**_**0**_ **: r = 1, H**_**a**_ **: r = 2)**		
LM	23.716	83.132	88.235
	(0.000)	(0.000)	(0.000)
LMF	5.566	20.340	21.667
	(0.000)	(0.001)	(0.000)
LRT	23.907	85.552	90.967
	(0.000)	(0.000)	(0.000)
	**Linearity test (H**_**0**_ **: r = 2, H**_**a**_ **: r = 3)**		
LM	4.039	2.865	4.305
	(0.401)	(0.581)	(0.366)
LMF	0.933	0.661	0.994
	(0.444)	(0.619)	(0.410)
LRT	4.045	2.868	4.311
	(0.400)	(0.580)	(0.366)

Notes:

*, **, and ***, respectively, denotes the statistical significance at 10, 5, and 1 per cent level. P-values are reported in parentheses. All possible test statistics are reported. The null hypothesis of Pesaran and Yamagata [30] is that the slope coefficients are homogeneous.

Results from Table 4 reject the assumption of homogenous coefficients across countries in our sample. These findings imply that the effects of institutional quality on financial inclusion are not homogenous. These important findings align with Karikari’s [31] and Hechmy’s [32] studies, which consider that products and services offered by banks and financial institutions in different environments will differ. Consequently, the operating environment, including institutions, legal systems, and other factors, affects the financial sector differently. Our findings also align with the results of previous studies indicating that environmental factors cause the lack of access to financial services and inefficient financial allocation [12, 26]. These effects are potentially affected by income level.

In response to these potential effects, countries are divided into three distinct groups (or regimes) because the effects of institutional quality on financial inclusion may depend upon the income level. These groups include (i) low-income countries, (ii) middle-income countries, and (iii) high-income countries. Table 5 presents the asymmetric effects of institutional quality on financial inclusion using the PSTR estimators for each of the proxies for institutional quality, including (i) the rule of law, (ii) government effectiveness, and (iii) regulatory quality.

**Table 5:** The asymmetric effects of institutional quality on financial inclusion using the PSTR estimators.

		Proxies for institutional quality		
Independent variables	Coefficients	The rule of law	Government effectiveness	Regulatory quality
Inflation	*β*_1_	0.0011**	0.0012**	0.0004
	(low-income)	(2.048)	(2.085)	(-2.731)
	*β*_2_	8.880***	0.083***	0.0085***
	(middle-income)	(2.734)	(2.769)	(3.475)
	*β*_3_	-8.986***	-7.437e+06***	-0.0011*
	(high-income)	(-2.731)	(-2.557)	(-1.926)
Urbanization	*β*_1_	1.678***	1.729***	1.470***
	(low-income)	(8.532)	(8.199)	(7.523)
	*β*_2_	3.618e+3***	35.283***	1.982***
	(middle-income)	(7.550)	(8.898)	(9.966)
	*β*_3_	-3.666e+3***	-3.539e+9***	-0.732***
	(high-income)	(-7.539)	(-7.687)	(-7.313)
Income per capita	*β*_1_	0.1145	0.197	0.0751
	(low-income)	(0.528)	(0.888)	(0.467)
	*β*_2_	-4.674e+3***	-44.119***	-2.393***
	(middle-income)	(-8.151)	(-9.209)	(-10.375)
	*β*_3_	4.737e+3***	-4.471e+9***	1.040***
	(high-income)	(8.141)	(-8.141)	(8.104)
	*β*_1_	-0.024*	-0.0027	-0.0067
The rule of law	(low-income)	(-1.951)	(-0.164)	(-0.733)
	*β*_2_	228.80***	1.042	0.1025***
Government effectiveness	(middle-income)	(3.038)	(1.472)	(3.905)
Regulatory quality	*β*_3_	232.485***	1.273e+8*	0.0295**
	(high-income)	(3.046)	(1.727)	(2.068)
Trans. speed 1	*γ*_1_	9.872	11.658	39.050
Centre of mass 1	*c*_1_	2.541	2.478	2.16
Trans. speed 2	*γ*_2_	9.730	7.370	21.16
Center of mass 2	*c*_2_	2.543	5.072	2.36

Table 5 provides empirical findings on the asymmetric effects of institutional quality on financial inclusion at different income levels. Our estimated coefficients *β*_1_, *β*_2_, *and β*_3_ represent the asymmetric effects of institutional quality on financial inclusion for low-income, middle-income, and high-income countries. We find that institutional quality generally enhances financial inclusion in the middle-income and high-income countries. Improving institutional quality results in an improvement in financially inclusive growth in these middle-and-high-income countries. However, the effect of institutional quality on financial inclusion cannot be confirmed in low-income countries. These findings imply that low-income countries may not benefit from institutional reform to support financial inclusion. Our findings align with previous studies, including Andrianova et al. [8], Love & Peria [23] and Ezirim et al. [33]. This finding calls for attention from the governments of low-income countries as institutional reform may cause loopholes during the reform process. Corruption emerges and negatively affects an effort to enhance financial inclusion through institutional reforms.

Our empirical results indicate that inflation supports financial inclusion in low-income and middle-income countries. The effect is the opposite for high-income countries. These findings imply that economic growth, which is linked with high inflation, supports financial inclusion. We also find that urbanization enhances financial inclusion for low-income and middle-income countries. Urbanization provides firms and individuals with access to different financial services and products. In emerging markets, financial services and products are very limited in rural areas compared to urban areas. Our empirical evidence also indicates that the effect of per capita income on financial inclusion is insignificant in low-income countries. However, this effect is negative for middle-income countries. In contrast, an increased GDP per capita supports financial inclusion in high-income countries. The effects of GDP per capita across three different groups of countries provide an interesting interpretation. For middle-income countries, when access to financial services and products becomes widely available, individuals limit the use of these services and products. However, for high-income countries, the banking system is well-developed, and the system’s reputation is well-tested. An increased income further supports more financially inclusive economic growth. Our findings are similar to those reported in previous studies, including Cihak et al. [34], Kim et al. [35], Levine et al. [36], Sarma and Pais [37] and Van et al. [38].

## Conclusions and policy implications

This study examines the asymmetric effects of institutional quality on financial inclusion for 110 countries from 2004 to 2020. We construct the index of financial inclusion (IFI) using the principal components analysis (PCA) to limit the correlations among the IFI constituents while preserving the index’s representativeness and comparability. Four indices are used as proxies for two important aspects of financial inclusion: (i) financial outreach and (ii) financial usage. The four indices are (i) outstanding debt with the commercial bank as a percentage of GDP, (ii) outstanding loan with commercial banks as a percentage of GDP, (iii) the number of commercial bank branches per 100,000 adults and (iv) the number of ATMs per 100,000 adults. We impose the 85 per cent threshold upon the cumulative variance contribution based on findings from previous empirical analyses. The PSTR is superior for estimating these asymmetric effects. We divide our sample into three distinct regimes, including (i) low-income countries, (ii) middle-income countries, and (iii) high-income countries. The income effects on financial inclusion are confirmed in our analysis.

Our results indicate that institutional quality enhances financial inclusion in middle-income and high-income countries. However, this positive effect cannot be confirmed in low-income countries. Inflation, urbanization, and income per capita all play a role in supporting financial inclusion. Supporting urbanization and improving per-capita income improve financial inclusion. For the middle-income and high-income countries, we recognize that institutional quality goes hand in hand with financial inclusion. This finding indicates that citizens can radically benefit from the reform of financial institutions and services. Such a superior institutional quality reflects consistency, high performance and connection between financial institutions and relevant state administrative agencies, thereby implying the advancement of the whole financial and legal system. Meanwhile, institutional quality is inversely correlated to financial inclusion in low-income countries. In other words, the reform of institutional quality in low-income countries inadvertently widens the gap in access to financial products and services among citizens. This finding can be explained by the typical demographic characteristics that cannot align with the progress of the financial system in low-income countries. In detail, people in these countries lack limited knowledge, education, financial literacy, and wealth, leading to a lack of collateral or credit scores for personal loans.

Policy implications have emerged based on these findings. Low-income countries can refer to the models of financial institutions successfully applied in high-income countries. For instance, governments should avoid direct credit schemes and government-subsidized first-tier lending programs to improve the attractiveness of the business environment. The governments can also remove the interest rate caps that prevent financial service providers, particularly those serving rural and remote communities, from recovering their total costs. On the other hand, the governments in these low-income countries can support people living in rural areas. Moreover, prudential regulation for deposit-taking financial intermediaries is essentially considered to support savings mobilization. For remote areas, governments should link rural finance to the growth of the wider financial system. It is crucial to address the financial literacy problems in the rural areas. The government should improve financial literacy in rural communities by including financial education in schools, training curricula, and promoting a savings and insurance culture among rural residents through public awareness campaigns and experience sharing among insured and uninsured people. Regarding an efficient legal system, policymakers should concentrate on improving legal institutions, which are critical to the financial system for a free flow of information, contract enforcement, and the protection of property rights. They should also consider the tax incentives to encourage individuals and corporations to get involved in actions that benefit society, such as job creation to eliminate poverty.

Enhancing financial inclusion also needs support from trade liberalization and deregulation. Reducing paperwork requirements for financial services such as account opening decreases bureaucracy. Furthermore, governments should limit their banking and financing sector intervention to promote financial independence. This practice encourages the independent central banks’ and financial institutions’ oversight and regulations to enforce contractual commitments and combat fraud. Credit is also distributed on a more market-based basis. The governments have limited influence over financial institutions. As a result, people and businesses have access to a wider range of financial services. Extending credit, accepting deposits, and conducting transactions in foreign currencies are made easier for banks. Foreign financial institutions have more freedom to operate and are considered equal to local financial firms.

Our study exhibits limitations. Dividing countries into different groups purely based on their income level may not be optimal because country-level characteristics may not be fully reflected in the income level. The current study also ignores culture and other socioeconomic characteristics, such as the level of financial literacy. As such, when data is available, studies in the future may need to consider these aspects to provide more comprehensive and convincing empirical evidence for policy implications to governments globally.

## Supporting information

S1 Appendix Table A1. The descriptive statistics of the variables. (DOCX)
