# Supplementary material for: Does institutional quality matter for financial inclusion? International evidence
Source: PLoS One. 2024 Feb 2;19(2):e0297431. doi: 10.1371/journal.pone.0297431 (PMC10836707; doi:10.1371/journal.pone.0297431)
Supplement: S1 Appendix — (DOCX) [file pone.0297431.s001.docx]

**Appendix**

**Table A1.** The descriptive statistics of the variables

| **Country name** | **Inflation** | **IFI** | **GDPP** | **Population** | **Government effectiveness** | **The rule of law** | **Regulatory quality** |
| --- | --- | --- | --- | --- | --- | --- | --- |
| Afghanistan | 6.12 | 0.11 | 7.55 | 15.82 | -1.36 | -1.70 | -1.39 |
|  | (7.45) | (0.01) | (0.2) | (0.18) | (0.15) | (0.15) | (0.24) |
| Angola | 11.09 | 0.14 | 8.90 | 16.52 | -1.12 | -1.23 | -1.04 |
|  | (23.69) | (0.03) | (0.11) | (0.23) | (0.12) | (0.14) | (0.11) |
| Albania | 2.30 | 0.22 | 9.30 | 14.26 | -0.23 | -0.50 | 0.13 |
|  | (0.70) | (0.04) | (0.18) | (0.08) | (0.22) | (0.14) | (0.18) |
| United Arab Emi | 2.37 | 0.40 | 11.13 | 15.69 | 1.13 | 0.55 | 0.75 |
|  | (3.44) | (0.08) | (0.17) | (0.3) | (0.28) | (0.2) | (0.24) |
| Armenia | 3.85 | 0.17 | 9.22 | 14.43 | -0.13 | -0.36 | 0.26 |
|  | (2.95) | (0.08) | (0.19) | (0.02) | (0.1) | (0.14) | (0.07) |
| Australia | 2.39 | 0.49 | 10.74 | 16.77 | 1.69 | 1.77 | 1.79 |
|  | (0.83) | (0.04) | (0.05) | (0.08) | (0.12) | (0.05) | (0.1) |
| Austria | 1.89 | 0.15 | 10.88 | 15.41 | 1.64 | 1.86 | 1.51 |
|  | (0.76) | (0.03) | (0.04) | (0.03) | (0.15) | (0.05) | (0.09) |
| Azerbaijan | 7.28 | 0.13 | 9.43 | 15.41 | -0.52 | -0.76 | -0.38 |
|  | (5.94) | (0.03) | (0.28) | (0.08) | (0.28) | (0.14) | (0.12) |
| Belgium | 1.94 | 0.35 | 10.79 | 16.19 | 1.49 | 1.38 | 1.30 |
|  | (1.16) | (0.03) | (0.03) | (0.03) | (0.23) | (0.07) | (0.07) |
| Bangladesh | 7.10 | 0.15 | 8.05 | 17.67 | -0.77 | -0.80 | -0.92 |
|  | (1.65) | (0.02) | (0.24) | (0.17) | (0.06) | (0.12) | (0.09) |
| Bulgaria | 3.63 | 0.28 | 9.80 | 15.49 | 0.15 | -0.07 | 0.61 |
|  | (3.59) | (0.06) | (0.15) | (0.01) | (0.12) | (0.04) | (0.05) |
| Bosnia and Herz | 1.54 | 0.27 | 9.36 | 14.32 | -0.60 | -0.32 | -0.19 |
|  | (2.64) | (0.02) | (0.15) | (0.02) | (0.13) | (0.13) | (0.15) |
| Bolivia | 5.20 | 0.19 | 8.86 | 15.74 | -0.55 | -1.01 | -0.82 |
|  | (3.21) | (0.06) | (0.15) | (0.1) | (0.13) | (0.16) | (0.18) |
| Brazil | 5.63 | 0.31 | 9.57 | 18.94 | -0.16 | -0.20 | -0.03 |
|  | (1.73) | (0.01) | (0.08) | (0.06) | (0.12) | (0.15) | (0.14) |
| Brunei Darussal | 0.36 | 0.23 | 11.10 | 12.61 | 0.93 | 0.55 | 0.93 |
|  | (0.82) | (0.03) | (0.06) | (0.08) | (0.25) | (0.21) | (0.2) |
| Botswana | 6.46 | 0.18 | 9.63 | 14.06 | 0.49 | 0.61 | 0.52 |
|  | (3.07) | (0.01) | (0.13) | (0.16) | (0.09) | (0.07) | (0.08) |
| Switzerland | 0.37 | 0.55 | 11.08 | 15.58 | 1.99 | 1.86 | 1.67 |
|  | (0.87) | (0.06) | (0.04) | (0.05) | (0.1) | (0.08) | (0.12) |
| Chile | 3.16 | 0.28 | 9.97 | 16.53 | 1.15 | 1.27 | 1.42 |
|  | (1.93) | (0.03) | (0.11) | (0.06) | (0.12) | (0.12) | (0.09) |
| Cameroon | 2.20 | 0.05 | 8.08 | 16.22 | -0.84 | -1.08 | -0.84 |
|  | (1.47) | (0.01) | (0.07) | (0.18) | (0.07) | (0.08) | (0.07) |
| Colombia | 4.33 | 0.19 | 9.44 | 17.40 | -0.06 | -0.40 | 0.28 |
|  | (1.59) | (0.03) | (0.14) | (0.08) | (0.1) | (0.12) | (0.16) |
| Comoros | 2.86 | 0.06 | 7.98 | 12.22 | -1.65 | -1.00 | -1.30 |
|  | (2.86) | (0.01) | (0.03) | (0.13) | (0.09) | (0.08) | (0.2) |
| Cabo Verde | 1.77 | 0.31 | 8.72 | 12.65 | 0.12 | 0.51 | -0.15 |
|  | (2.41) | (0.05) | (0.11) | (0.11) | (0.14) | (0.1) | (0.13) |
| Costa Rica | 6.23 | 0.25 | 9.71 | 15.03 | 0.31 | 0.50 | 0.48 |
|  | (4.60) | (0.03) | (0.13) | (0.13) | (0.12) | (0.05) | (0.06) |
| Cyprus | 1.22 | 0.44 | 10.52 | 13.53 | 1.24 | 1.01 | 1.16 |
|  | (1.9) | (0.04) | (0.06) | (0.05) | (0.23) | (0.17) | (0.17) |
| Czech Republic | 2.14 | 0.26 | 10.45 | 15.85 | 0.95 | 1.00 | 1.14 |
|  | (1.46) | (0.03) | (0.09) | (0.01) | (0.07) | (0.11) | (0.11) |
| Germany | 1.46 | 0.38 | 10.80 | 17.96 | 1.61 | 1.68 | 1.62 |
|  | (0.66) | (0.01) | (0.07) | (0.01) | (0.08) | (0.08) | (0.11) |
| Denmark | 1.47 | 0.30 | 10.87 | 15.40 | 2.05 | 1.94 | 1.76 |
|  | (0.91) | (0.05) | (0.04) | (0.03) | (0.19) | (0.07) | (0.12) |
| Dominican Repub | 7.43 | 0.13 | 9.50 | 15.81 | -0.51 | -0.58 | -0.16 |
|  | (12.06) | (0.02) | (0.19) | (0.12) | (0.13) | (0.15) | (0.12) |
| Ecuador | 3.12 | 0.17 | 9.29 | 16.08 | -0.63 | -0.96 | -1.01 |
|  | (2.15) | (0.02) | (0.09) | (0.09) | (0.24) | (0.22) | (0.16) |
| Egypt, Arab Rep | 11.95 | 0.20 | 9.22 | 17.42 | -0.55 | -0.35 | -0.55 |
|  | (5.82) | (0.01) | (0.1) | (0.09) | (0.2) | (0.22) | (0.27) |
| Spain | 1.80 | 0.60 | 10.55 | 17.40 | 1.08 | 1.07 | 1.07 |
|  | (1.51) | (0.1) | (0.04) | (0.04) | (0.17) | (0.1) | (0.17) |
| Estonia | 3.24 | 0.33 | 10.30 | 13.72 | 1.06 | 1.19 | 1.47 |
|  | (2.77) | (0.04) | (0.12) | (0.01) | (0.08) | (0.11) | (0.14) |
| Finland | 1.37 | 0.23 | 10.74 | 15.33 | 2.07 | 1.99 | 1.77 |
|  | (1.24) | (0.02) | (0.03) | (0.03) | (0.15) | (0.05) | (0.11) |
| Georgia | 5.06 | 0.21 | 9.27 | 14.57 | 0.31 | -0.07 | 0.56 |
|  | (3.51) | (0.09) | (0.24) | (0.02) | (0.4) | (0.36) | (0.54) |
| Ghana | 12.55 | 0.20 | 8.31 | 16.40 | -0.10 | 0.02 | -0.04 |
|  | (3.89) | (0.01) | (0.2) | (0.18) | (0.13) | (0.08) | (0.14) |
| Guinea | 15.35 | 0.02 | 7.61 | 15.10 | -1.09 | -1.35 | -1.01 |
|  | (8.89) | (0.02) | (0.12) | (0.15) | (0.14) | (0.12) | (0.15) |
| Greece | 1.54 | 0.35 | 10.37 | 15.94 | 0.48 | 0.51 | 0.60 |
|  | (2.06) | (0.03) | (0.11) | (0.01) | (0.17) | (0.3) | (0.26) |
| Honduras | 5.80 | 0.22 | 8.53 | 15.32 | -0.66 | -0.97 | -0.36 |
|  | (2.23) | (0.02) | (0.08) | (0.15) | (0.09) | (0.12) | (0.14) |
| Croatia | 1.90 | 0.34 | 10.12 | 14.67 | 0.55 | 0.22 | 0.48 |
|  | (1.77) | (0.05) | (0.06) | (0.01) | (0.09) | (0.12) | (0.07) |
| Hungary | 3.58 | 0.23 | 10.18 | 15.74 | 0.64 | 0.69 | 0.93 |
|  | (2.38) | (0.02) | (0.09) | (0.02) | (0.13) | (0.2) | (0.23) |
| Indonesia | 6.13 | 0.18 | 9.08 | 18.64 | -0.18 | -0.54 | -0.30 |
|  | (2.86) | (0.04) | (0.19) | (0.13) | (0.2) | (0.17) | (0.18) |
| India | 7.04 | 0.20 | 8.42 | 19.79 | 0.00 | 0.01 | -0.34 |
|  | (2.79) | (0.03) | (0.25) | (0.12) | (0.14) | (0.08) | (0.09) |
| Ireland | 1.16 | 0.39 | 11.03 | 14.85 | 1.47 | 1.65 | 1.68 |
|  | (2.27) | (0.08) | (0.18) | (0.07) | (0.13) | (0.14) | (0.13) |
| Iran, Islamic R | 18.57 | 0.24 | 9.48 | 17.80 | -0.47 | -0.87 | -1.44 |
|  | (9.85) | (0.06) | (0.06) | (0.1) | (0.15) | (0.14) | (0.17) |
| Iraq | 9.87 | 0.14 | 9.16 | 16.91 | -1.31 | -1.62 | -1.24 |
|  | (16.62) | (0.01) | (0.14) | (0.15) | (0.2) | (0.16) | (0.17) |
| Iceland | 4.68 | 0.45 | 10.83 | 12.62 | 1.63 | 1.75 | 1.30 |
|  | (3.34) | (0.08) | (0.07) | (0.06) | (0.21) | (0.12) | (0.21) |
| Italy | 1.50 | 0.37 | 10.66 | 17.53 | 0.43 | 0.40 | 0.85 |
|  | (1.05) | (0.03) | (0.04) | (0.03) | (0.11) | (0.09) | (0.14) |
| Jamaica | 8.80 | 0.17 | 9.18 | 14.24 | 0.24 | -0.33 | 0.22 |
|  | (5.13) | (0.02) | (0.03) | (0.04) | (0.17) | (0.09) | (0.06) |
| Jordan | 3.70 | 0.38 | 9.25 | 15.71 | 0.11 | 0.32 | 0.18 |
|  | (3.53) | (0.01) | (0.06) | (0.26) | (0.08) | (0.09) | (0.11) |
| Japan | 0.29 | 0.44 | 10.56 | 18.56 | 1.56 | 1.40 | 1.20 |
|  | (0.93) | (0.03) | (0.04) | (0.02) | (0.16) | (0.12) | (0.12) |
| Kenya | 9.81 | 0.11 | 8.16 | 16.17 | -0.49 | -0.72 | -0.24 |
|  | (5.51) | (0.02) | (0.12) | (0.21) | (0.13) | (0.24) | (0.06) |
| Cambodia | 5.15 | 0.16 | 8.00 | 14.94 | -0.82 | -1.09 | -0.49 |
|  | (5.87) | (0.09) | (0.24) | (0.15) | (0.15) | (0.1) | (0.08) |
| Korea, Rep. | 2.24 | 0.58 | 10.47 | 17.52 | 1.12 | 1.02 | 0.95 |
|  | (1.21) | (0.05) | (0.14) | (0.03) | (0.12) | (0.11) | (0.14) |
| Kuwait | 3.60 | 0.29 | 11.02 | 14.97 | 0.02 | 0.36 | 0.11 |
|  | (2.33) | (0.05) | (0.14) | (0.23) | (0.14) | (0.23) | (0.21) |
| Lao PDR | 4.62 | 0.13 | 8.57 | 14.49 | -0.77 | -0.93 | -0.97 |
|  | (2.95) | (0.04) | (0.27) | (0.17) | (0.24) | (0.12) | (0.22) |
| Lebanon | 2.93 | 0.53 | 9.70 | 15.40 | -0.41 | -0.69 | -0.20 |
|  | (3.11) | (0.03) | (0.1) | (0.17) | (0.17) | (0.17) | (0.14) |
| Lesotho | 5.60 | 0.10 | 7.78 | 13.15 | -0.50 | -0.23 | -0.53 |
|  | (1.91) | (0.02) | (0.15) | (0.11) | (0.23) | (0.07) | (0.12) |
| Lithuania | 2.95 | 0.24 | 10.22 | 14.53 | 0.85 | 0.83 | 1.09 |
|  | (2.75) | (0.02) | (0.19) | (0.06) | (0.17) | (0.14) | (0.1) |
| Luxembourg | 1.87 | 0.33 | 11.60 | 13.06 | 1.70 | 1.81 | 1.71 |
|  | (1.01) | (0.03) | (0.04) | (0.12) | (0.08) | (0.05) | (0.07) |
| Latvia | 3.95 | 0.31 | 10.10 | 14.16 | 0.80 | 0.80 | 1.05 |
|  | (4.3) | (0.05) | (0.15) | (0.06) | (0.21) | (0.12) | (0.08) |
| Moldova | 7.64 | 0.24 | 9.13 | 14.00 | -0.62 | -0.39 | -0.15 |
|  | (3.99) | (0.02) | (0.2) | (0.03) | (0.15) | (0.07) | (0.15) |
| Madagascar | 9.01 | 0.05 | 7.36 | 15.79 | -0.92 | -0.69 | -0.52 |
|  | (3.37) | (0.01) | (0.03) | (0.22) | (0.31) | (0.24) | (0.21) |
| Mexico | 4.14 | 0.15 | 9.83 | 18.32 | 0.14 | -0.52 | 0.31 |
|  | (0.89) | (0.02) | (0.04) | (0.08) | (0.15) | (0.1) | (0.11) |
| North Macedonia | 1.71 | 0.24 | 9.51 | 13.99 | -0.04 | -0.27 | 0.28 |
|  | (2.31) | (0.05) | (0.13) | (0.01) | (0.14) | (0.11) | (0.23) |
| Malta | 1.90 | 0.42 | 10.44 | 12.91 | 1.05 | 1.31 | 1.21 |
|  | (1.05) | (0.02) | (0.14) | (0.07) | (0.17) | (0.21) | (0.13) |
| Montenegro | 2.73 | 0.30 | 9.75 | 12.90 | 0.10 | -0.02 | 0.05 |
|  | (2.4) | (0.05) | (0.13) | (0.03) | (0.15) | (0.11) | (0.21) |
| Mongolia | 9.48 | 0.30 | 9.07 | 14.44 | -0.44 | -0.29 | -0.24 |
|  | (5.99) | (0.07) | (0.27) | (0.12) | (0.17) | (0.1) | (0.14) |
| Mozambique | 8.15 | 0.09 | 6.98 | 15.90 | -0.67 | -0.74 | -0.54 |
|  | (5.31) | (0.03) | (0.17) | (0.2) | (0.14) | (0.2) | (0.14) |
| Mauritania | 5.15 | 0.56 | 8.50 | 14.37 | -0.81 | -0.81 | -0.64 |
|  | (3.03) | (0.12) | (0.05) | (0.23) | (0.22) | (0.15) | (0.22) |
| Mauritius | 4.33 | 0.38 | 9.77 | 13.16 | 0.85 | 0.89 | 0.85 |
|  | (2.84) | (0.04) | (0.17) | (0) | (0.13) | (0.1) | (0.23) |
| Malaysia | 2.34 | 0.38 | 10.00 | 16.84 | 1.04 | 0.49 | 0.60 |
|  | (1.3) | (0.03) | (0.15) | (0.12) | (0.12) | (0.07) | (0.14) |
| Namibia | 5.52 | 0.28 | 9.13 | 13.75 | 0.12 | 0.19 | 0.02 |
|  | (1.92) | (0.08) | (0.1) | (0.2) | (0.06) | (0.12) | (0.11) |
| Nicaragua | 7.34 | 0.14 | 8.52 | 15.04 | -0.83 | -0.78 | -0.44 |
|  | (4.05) | (0.02) | (0.12) | (0.08) | (0.1) | (0.15) | (0.14) |
| Netherlands | 1.60 | 0.34 | 10.88 | 16.50 | 1.82 | 1.83 | 1.81 |
|  | (0.72) | (0.03) | (0.04) | (0.06) | (0.09) | (0.06) | (0.12) |
| Norway | 2.00 | 0.26 | 11.03 | 15.19 | 1.90 | 1.97 | 1.56 |
|  | (0.93) | (0.02) | (0.02) | (0.07) | (0.07) | (0.05) | (0.17) |
| Nepal | 7.20 | 0.22 | 7.83 | 15.35 | -0.89 | -0.71 | -0.70 |
|  | (2.74) | (0.05) | (0.18) | (0.12) | (0.09) | (0.13) | (0.11) |
| Pakistan | 8.84 | 0.19 | 8.32 | 17.99 | -0.65 | -0.82 | -0.64 |
|  | (4.43) | (0.01) | (0.08) | (0.13) | (0.14) | (0.09) | (0.09) |
| Panama | 2.81 | 0.44 | 10.06 | 14.71 | 0.13 | -0.09 | 0.37 |
|  | (2.48) | (0.04) | (0.24) | (0.11) | (0.11) | (0.08) | (0.09) |
| Peru | 2.86 | 0.17 | 9.24 | 16.94 | -0.30 | -0.57 | 0.39 |
|  | (1.14) | (0.07) | (0.2) | (0.06) | (0.19) | (0.09) | (0.15) |
| Philippines | 3.90 | 0.14 | 8.77 | 17.60 | 0.02 | -0.46 | -0.10 |
|  | (1.93) | (0.03) | (0.19) | (0.09) | (0.11) | (0.09) | (0.09) |
| Poland | 2.08 | 0.26 | 10.12 | 16.96 | 0.60 | 0.60 | 0.91 |
|  | (1.6) | (0.04) | (0.18) | (0.01) | (0.14) | (0.16) | (0.1) |
| Portugal | 1.47 | 0.56 | 10.37 | 15.67 | 1.09 | 1.10 | 0.94 |
|  | (1.31) | (0.05) | (0.04) | (0.04) | (0.13) | (0.08) | (0.18) |
| Russia | 8.53 | 0.31 | 10.10 | 18.48 | -0.30 | -0.82 | -0.37 |
|  | (3.71) | (0.09) | (0.1) | (0.01) | (0.19) | (0.09) | (0.12) |
| Rwanda | 6.88 | 0.07 | 7.37 | 14.39 | -0.10 | -0.28 | -0.21 |
|  | (4.73) | (0.02) | (0.22) | (0.13) | (0.29) | (0.35) | (0.38) |
| Saudi Arabia | 2.81 | 0.23 | 10.74 | 16.97 | 0.01 | 0.11 | 0.05 |
|  | (2.88) | (0.04) | (0.04) | (0.14) | (0.24) | (0.08) | (0.08) |
| Singapore | 1.81 | 0.48 | 11.30 | 15.44 | 2.20 | 1.72 | 1.96 |
|  | (2.08) | (0.05) | (0.14) | (0.11) | (0.12) | (0.1) | (0.19) |
| El Salvador | 2.34 | 0.23 | 8.95 | 15.24 | -0.22 | -0.66 | 0.16 |
|  | (2.21) | (0.01) | (0.08) | (0.08) | (0.15) | (0.14) | (0.19) |
| Serbia | 6.85 | 0.22 | 9.60 | 15.20 | -0.06 | -0.36 | -0.11 |
|  | (4.67) | (0.03) | (0.12) | (0.01) | (0.15) | (0.23) | (0.24) |
| Slovak | 2.45 | 0.25 | 10.19 | 14.90 | 0.83 | 0.54 | 0.99 |
|  | (2.11) | (0.03) | (0.15) | (0.01) | (0.07) | (0.05) | (0.12) |
| Slovenia | 1.94 | 0.36 | 10.44 | 13.90 | 1.04 | 1.00 | 0.75 |
|  | (1.53) | (0.04) | (0.07) | (0.03) | (0.09) | (0.07) | (0.13) |
| Sweden | 1.16 | 0.25 | 10.81 | 15.92 | 1.91 | 1.94 | 1.74 |
|  | (1.14) | (0.02) | (0.05) | (0.06) | (0.1) | (0.06) | (0.14) |
| Seychelles | 6.43 | 0.27 | 10.04 | 10.79 | 0.27 | 0.10 | -0.39 |
|  | (11.21) | (0.03) | (0.16) | (0.09) | (0.17) | (0.08) | (0.26) |
| Chad | 2.29 | 0.02 | 7.41 | 14.84 | -1.46 | -1.41 | -1.11 |
|  | (5.58) | (0.01) | (0.07) | (0.18) | (0.11) | (0.14) | (0.1) |
| Thailand | 2.17 | 0.33 | 9.61 | 17.21 | 0.31 | -0.08 | 0.22 |
|  | (1.93) | (0.04) | (0.14) | (0.13) | (0.08) | (0.12) | (0.09) |
| Trinidad | 6.15 | 0.23 | 10.22 | 13.49 | 0.25 | -0.13 | 0.34 |
|  | (3.13) | (0.03) | (0.07) | (0.01) | (0.09) | (0.06) | (0.28) |
| Turkey | 9.39 | 0.25 | 10.02 | 17.79 | 0.21 | -0.02 | 0.24 |
|  | (2.79) | (0.05) | (0.17) | (0.11) | (0.14) | (0.16) | (0.14) |
| Uganda | 7.03 | 0.10 | 7.53 | 15.74 | -0.54 | -0.38 | -0.22 |
|  | (4.05) | (0) | (0.13) | (0.28) | (0.06) | (0.1) | (0.07) |
| Ukraine | 13.20 | 0.24 | 9.39 | 17.26 | -0.59 | -0.76 | -0.49 |
|  | (11.15) | (0.04) | (0.06) | (0.01) | (0.15) | (0.04) | (0.12) |
| Vietnam | 7.65 | 0.33 | 8.60 | 17.15 | -0.16 | -0.36 | -0.54 |
|  | (5.85) | (0.05) | (0.24) | (0.15) | (0.15) | (0.24) | (0.12) |
| Zambia | 10.58 | 0.09 | 8.02 | 15.56 | -0.69 | -0.40 | -0.51 |
|  | (4.22) | (0.01) | (0.15) | (0.2) | (0.15) | (0.11) | (0.08) |
| Zimbabwe | 1.07 | 0.21 | 7.84 | 15.28 | -1.28 | -1.60 | -1.89 |
|  | (2.32) | (0.07) | (0.18) | (0.05) | (0.15) | (0.23) | (0.25) |

*Notes*: The descriptive statistics of all included variables. The standard deviation is reported in parentheses.
